# Associations of Person-Centred Care, Organisational Communication Satisfaction, and Ethical Climate with Job Involvement Among Intensive Care Unit Nurses in South Korea: A Cross-Sectional Study

**DOI:** 10.3390/healthcare14213824

**Published:** 2026-11-09

**Authors:** Sooyeon Kwak, Younjae Oh

**Affiliations:** 1 Graduate School of Nursing Science, Hallym University, Hallymdaehak-gil 1, Chuncheon 24252, Republic of Korea; tndus0126@hallym.or.kr; 2 Hallym University Chuncheon Sacred Heart Hospital, 77, Sakju-ro, Chuncheon 24253, Republic of Korea; 3 Research Institute of Nursing Science, College of Nursing, Hallym University, Hallymdaehak-gil 1, Chuncheon 24252, Republic of Korea

**Keywords:** job involvement, person-centred care, organisational communication satisfaction, ethical climate, intensive care nurses

## Abstract

**Highlights:**

**What are the main findings?**

- Among ICU nurses in South Korea, the vertical and horizontal communication subdomains of organisational communication satisfaction, alongside the prerequisites subdomain of person-centred care, were independently and positively associated with job involvement.
- The person-centred care subdomains accounted for an additional 8.7 percentage points of the variance in job involvement beyond the control variables, ethical climate, and organisational communication satisfaction.

**What are the implications of the main findings?**

- Communication with superiors and peers warrants consideration as a potential focus for future studies of ICU nurses’ job involvement.
- The prerequisites for person-centred care, including competence, interpersonal skills, and professional values, warrant further investigation in relation to job involvement.

**Abstract:**

**Background/Objectives**: Job involvement reflects nurses’ psychological identification with their jobs. This study examined the associations of person-centred care, organisational communication satisfaction, and ethical climate with job involvement among intensive care unit (ICU) nurses in South Korea. **Methods**: A cross-sectional descriptive correlational study was conducted in August 2025 among 217 ICU nurses from three general hospitals using self-administered questionnaires. Data were analysed using Pearson’s correlation coefficients and hierarchical multiple regression. Gender, educational level, and total clinical experience were entered first, followed by ethical climate, organisational communication satisfaction, and person-centred care subdomains. **Results**: Job involvement was positively correlated with person-centred care (*r* = 0.637), organisational communication satisfaction (*r* = 0.598), and ethical climate (*r* = 0.536; all *p* < 0.001). The final regression model explained 53.4% of the variance in job involvement (*R*^2^ = 0.534; adjusted *R*^2^ = 0.491), with person-centred care subdomains accounting for an additional 8.7 percentage points (Δ*R*^2^ = 0.087). The vertical (β = 0.385) and horizontal (β = 0.209) communication subdomains, along with the prerequisites subdomain of person-centred care (β = 0.191), demonstrated independent positive associations. The communication media subdomain (β = −0.166) showed a negative coefficient despite its positive bivariate association, warranting cautious interpretation. **Conclusions**: The findings highlight the relevance of nurses’ communication experiences with superiors and peers, alongside the professional attributes that support person-centred practice, in understanding job involvement in ICU settings. Longitudinal and interventional studies are needed to clarify the temporal nature of these associations and evaluate organisational implications.

## 1. Introduction

Intensive care units (ICUs) are complex and dynamic clinical environments wherein critically ill patients receive continuous monitoring and highly specialised treatment. In this context, ICU nurses are required to rapidly recognise changing patient conditions and make timely clinical judgements [1,2]. The demanding nature of ICU nursing also makes nurses’ psychological identification with their jobs a relevant area of inquiry. Kanungo defined job involvement as the degree to which individuals psychologically identify with their jobs and regard them as central to their self-concept [3]. Conceptually, job involvement has been linked to both individual attributes, including psychological resources and competencies, and situational conditions, such as job characteristics, organisational climate, supervisory support, and open communication [4,5]. From Kanungo’s perspective, these factors matter because individuals tend to become more involved in their jobs when they perceive them as having the potential to satisfy their salient intrinsic and extrinsic needs [3]. Among hospital nurses, higher job involvement has been positively correlated with nursing performance [6] and caring behaviours [7]. Given these associations and the clinical demands of intensive care, understanding ICU nurses’ job involvement requires consideration of both their professional attributes and care practices and the organisational conditions in which they work.

Among these practice-related and organisational factors, person-centred care may be related to nurses’ job involvement. Person-centred care involves recognising patients as unique individuals and providing individualised care that takes into account their values, preferences, needs, and lived experiences [8]. Although urgent life-sustaining tasks may take precedence over patients’ individual needs and the relational dimensions of care in the advanced technological environment of ICUs [9,10], person-centred care remains a vital nursing practice in intensive care [11,12]. It encompasses not only care processes but also prerequisite characteristics among nurses and the care environment [13,14]. As this approach requires nurses to respond to individual patients using their professional judgement and to provide relational care, it may allow them to exercise their competence and act on their professional values [15,16]. These experiences may contribute to the significance nurses attach to their work and their psychological identification with their jobs. Healthcare professionals providing person-centred care have reported meaningful work, stronger patient relationships, and job satisfaction [17]. Furthermore, person-centred care is negatively correlated with turnover intention among ICU nurses [11]. However, nurses’ ability to provide good care also depends on organisational support. Previous studies have identified organisational constraints, including unclear practice guidelines and one-way communication from management, that may hinder nurses’ responses to patients’ needs and their efforts to enact professional values [12,15].

Against this background, organisational communication is particularly relevant because it shapes how nurses exchange information, share professional knowledge, and express their perspectives within the workplace [18,19]. Organisational communication satisfaction reflects members’ satisfaction with information exchange, interactions with supervisors and peers, communication media, and the overall communication climate [20]. Effective organisational communication facilitates clinical information exchange and coordination of care [18,21], while greater communication satisfaction has been associated with job satisfaction and organisational commitment among healthcare professionals [19,22]. Conversely, difficulties in communication between nurses and their organisations, including limited opportunities to express concerns and receive meaningful responses, have been associated with reduced work efficiency, poorer nursing performance, lower morale, a diminished sense of belonging, increased stress, burnout, and turnover intentions [15,23]. Satisfaction with organisational communication may be related to nurses’ perceptions that their professional contribution is recognised and valued, potentially reinforcing their psychological identification with their jobs.

Beyond communication, organisational conditions also shape whether nurses can act on the ethical values underlying their practice [16,24]. Ethical climate reflects members’ shared perceptions of what constitutes ethically appropriate behaviour and how ethical issues are addressed within an organisation [25]. This is particularly relevant in nursing because organisational conditions can enable or constrain nurses’ ability to practise in accordance with their professional and ethical values [26,27]. A more positive ethical climate has been linked with greater job satisfaction [28] and lower turnover intention [29]. Ethical climate was moderately and positively correlated with job involvement among hospital nurses [7]. In the context of ICUs—where nurses frequently encounter ethically complex situations involving life-sustaining treatment, end-of-life care, and patients’ wishes [16,30]—a supportive ethical climate may provide opportunities for nurses to deliver care consistent with their understanding of good nursing. When nurses perceive their professional values to be reflected in everyday clinical practice, they may experience greater meaning in their work and a stronger sense of psychological identification with their professional roles.

Although previous studies have linked person-centred care, organisational communication satisfaction, and ethical climate to nursing and work-related outcomes, to the best of our knowledge, few studies have examined these factors simultaneously in relation to job involvement among ICU nurses. These factors may also be interrelated: ethical climate includes nurses’ relationships with peers, managers, and physicians, potentially overlapping with aspects of organisational communication. Moreover, both communication and ethical climate form part of the organisational context in which person-centred care is delivered. Therefore, examining them separately fails to clarify whether their associations with job involvement persist after accounting for one another. In South Korean ICUs, hierarchical organisational norms and physician-centred decision-making may constrain nurses’ opportunities to voice concerns, provide individualised care, and enact their professional values [16,31]. Hence, understanding these factors’ associations with job involvement may help nurse managers identify aspects of organisational communication and professional development that warrant attention when supporting ICU nurses’ psychological identification with their jobs. Accordingly, this cross-sectional study aimed to examine the associations of person-centred care, organisational communication satisfaction, and ethical climate with job involvement among ICU nurses in South Korea.

## 2. Materials and Methods

### 2.1. Participants and Setting

This study employed a cross-sectional descriptive correlational design and is reported in accordance with the Strengthening the Reporting of Observational Studies in Epidemiology (STROBE) guidelines [32]. ICU nurses were recruited using convenience sampling from three general hospitals, each with more than 200 beds, located across three regions of South Korea: Seoul, Gangwon Province, and Gyeonggi Province. Staff nurses working in the ICUs who provided written informed consent were eligible to participate. Based on previous research, nurses with less than six months of ICU experience were excluded because they were still undergoing orientation and training and had not yet begun performing independent nursing duties [15]. Additionally, unit managers were excluded because their roles and work environments differ from those of staff nurses [33,34]. The required sample size was calculated using G*Power (version 3.1.9; Heinrich-Heine-Universität Düsseldorf, Düsseldorf, Germany) for the omnibus *F* test in multiple linear regression (fixed model, *R*^2^ deviation from zero). The original calculation assumed 23 predictors, a significance level of *α* = 0.05, statistical power of 0.90, and a medium effect size (*f*^2^ = 0.15), yielding a minimum sample size of 202. The 23 candidate predictors comprised the 18 predictors subsequently included in the final model, three dummy variables for age, one for marital status, and one for religion. Allowing for an approximately 15% non-response or incomplete questionnaire rate, 240 questionnaires were distributed. During the revision process, the sample size calculation was repeated using the same assumptions but specifying 18 predictors, which yielded a minimum required sample size of 183. The final sample of 217 exceeded both requirements. Importantly, these calculations relate to the overall regression model and do not demonstrate adequate statistical power for detecting individual regression coefficients.

Data collection was conducted between 1 and 31 August 2025. The first author visited the participating ICUs in person to distribute the self-administered questionnaires. Before obtaining written informed consent, the first author explained the study’s purpose and informed potential participants that participation was voluntary and that they could withdraw at any time without repercussions. Participants sealed their completed questionnaires in envelopes to ensure confidentiality, and the first author collected them during hospital visits. Of the 240 questionnaires distributed, 229 were returned (response rate = 95.4%). Twelve questionnaires were excluded due to item non-response or because the same response option was selected for every item. The remaining 217 questionnaires were included in the final analysis. The data were analysed as a pooled sample. Hospital-specific participant counts and findings are not reported because participants expressed that reporting results at the hospital level could compromise the confidentiality of the participating institutions.

### 2.2. Instruments

#### 2.2.1. General Characteristics

The questionnaire included seven items assessing participants’ general characteristics: age, gender, marital status, educational level, religion, total clinical experience, and ICU experience. Age and experience were grouped for descriptive and group-comparison analyses, following the categorical reporting approaches adopted in previous nursing ethics studies [11,35]. The age and experience categories were largely based on those used in previous studies of Korean ICU nurses, with a minor modification to the upper age boundary [11,31].

#### 2.2.2. Job Involvement

Job involvement was assessed using the instrument modified and adapted by Kang and Kwon [36] from the original tool developed by Kanungo [3]. The instrument consists of nine items, including eight positively worded items and one negatively worded item. Each item is rated on a five-point Likert scale ranging from 1 (strongly disagree) to 5 (strongly agree), with the negatively worded item reverse-coded. Higher scores indicate higher levels of job involvement. Cronbach’s α was 0.82 in Kang and Kwon’s study [36] and 0.72 in this study.

#### 2.2.3. Person-Centred Care

Person-centred care was assessed using the Korean version of the Person-Centred Practice Inventory–Staff (K-PCPI-S), validated by Kim and Tak [37] from the original PCPI-S developed by Slater et al. [38]. The instrument consists of 51 items across three subdomains: prerequisites (13 items), care environment (22 items), and care processes (16 items). Prerequisites concern nurses’ professional attributes, including professional competence, interpersonal skills, commitment to care, self-awareness, and clarity of beliefs and values. The care environment concerns the organisational and contextual conditions that support person-centred practice, whereas care processes concern how person-centred care is delivered through interactions with patients [37,38]. Each item is rated on a five-point Likert scale ranging from 1 (strongly disagree) to 5 (strongly agree), with higher scores reflecting higher levels of person-centred care. Cronbach’s α was 0.95 in Kim and Tak’s study [37] and 0.96 in this study. For the subdomains, Cronbach’s α coefficients in this study were 0.88 for prerequisites, 0.94 for care environment, and 0.93 for care processes.

#### 2.2.4. Organisational Communication Satisfaction

Organisational communication satisfaction was assessed using the Korean version of the Communication Satisfaction Questionnaire, originally developed by Downs and Hazen [20] and validated by Hong [39]. The instrument consists of 24 items across four subdomains: vertical communication (8 items), horizontal communication (5 items), communication media (6 items), and organisational climate (5 items). Each item is rated on a five-point Likert scale ranging from 1 (strongly disagree) to 5 (strongly agree). Higher scores indicate higher levels of organisational communication satisfaction. Cronbach’s α was 0.90 in Downs and Hazen’s study [20], 0.88 in Hong’s study [39], and 0.92 in this study. For the subdomains, Cronbach’s α coefficients in this study were 0.83 for vertical communication, 0.76 for horizontal communication, 0.62 for communication media, and 0.81 for organisational climate.

#### 2.2.5. Ethical Climate

Ethical climate was assessed using the Korean version of the Hospital Ethical Climate Survey (K-HECS), validated by Hwang and Park [40] from the original HECS developed by Olson [25]. The instrument consists of 26 items across five subdomains: peers (4 items), patients (4 items), managers (6 items), hospital (6 items), and physicians (6 items). Each item is rated on a five-point Likert scale ranging from 1 (almost never true) to 5 (almost always true). Higher scores indicate a more positive perception of the ethical climate. Cronbach’s α was 0.91 in Olson’s study [25], 0.95 in Hwang and Park’s study [40], and 0.93 in this study. For the subdomains, Cronbach’s α coefficients in this study were 0.77 for peers, 0.70 for patients, 0.96 for managers, 0.74 for hospital, and 0.93 for physicians.

### 2.3. Data Analysis

Data were analysed using descriptive statistics, Cronbach’s α reliability analysis, independent-sample *t*-tests, one-way analysis of variance (ANOVA) with post hoc Scheffé tests, Pearson’s correlation coefficients, and hierarchical multiple regression analysis. The hierarchical multiple regression was performed to examine the associations of ethical climate, organisational communication satisfaction, and person-centred care with job involvement. Categorical variables were dummy-coded, with female gender, graduate degree or higher, and ≥10 years of total clinical experience serving as the reference categories. Because the 217 questionnaires included in the analysis contained no missing item responses, no imputation was required. Scale and subscale scores were calculated as the mean of the item responses within the respective scale or subscale.

In the hierarchical multiple regression analysis, the six demographic and clinical dummy variables were entered first, followed by the five subdomains of ethical climate, four subdomains of organisational communication satisfaction, and three subdomains of person-centred care, yielding 18 predictors in the final model. The variables were entered in this order to examine the additional explanatory contribution of organisational communication satisfaction and person-centred care following the inclusion of ethical climate and demographic and clinical characteristics. This sequence progressed from the broader organisational context to communication experiences and person-centred practice. Because person-centred care also encompasses the care environment, this order reflects the conceptual organisation of the study variables rather than an assumed causal ordering or a strict distinction between organisational and individual factors.

Age and ICU experience were excluded from the final model to minimise conceptual overlap with total clinical experience. Marital status and religion were not retained because neither showed a significant univariate difference in job involvement; gender and educational level were retained based on findings from previous research [41,42]. Data from the three hospitals were analysed as a pooled sample. Hospital membership was not included as a covariate, and no multilevel or cluster-adjusted analysis was performed. The Durbin–Watson statistic does not assess or exclude within-hospital dependence. The full correlation matrix is provided in Table S2. No study-wide adjustment for multiple testing was applied; the Scheffé adjustment was used exclusively for post hoc comparisons following ANOVA.

Multicollinearity and autocorrelation were assessed using variance inflation factors (VIFs) and the Durbin–Watson statistic, respectively, with VIF values below 10 and Durbin–Watson values between 1.5 and 2.5 considered acceptable [43,44]. Unstandardised regression coefficients (*B*) in the final model are reported with 95% confidence intervals (CIs). All statistical tests were two-sided, and a *p*-value < 0.05 was considered statistically significant. All analyses were performed using IBM SPSS Statistics for Windows, version 29.0.2.0 (IBM Corp., Armonk, NY, USA).

### 2.4. Ethical Considerations

This study was conducted in accordance with the Declaration of Helsinki and was approved by the Institutional Review Board of Hallym University prior to data collection (HIRB No. 2025-064; date of approval: 29 July 2025). Written informed consent outlining the study’s purpose, confidentiality, and anonymity was obtained from all participants.

## 3. Results

### 3.1. Differences in Job Involvement According to Nurses’ General Characteristics

As shown in Table 1, job involvement differed significantly according to age, total clinical experience, and ICU experience. A significant difference was observed based on age (*F* = 4.586, *p* = 0.004). The Scheffé post hoc test revealed that nurses aged ≤25 years (Mean = 3.69, standard deviation *[SD]* = 0.38) had significantly higher job involvement than those aged 26–30 years (Mean = 3.46, *SD* = 0.44; *p* < 0.05).

Job involvement also differed significantly according to total clinical experience (*F* = 5.112, *p* = 0.002). The Scheffé post hoc test showed that nurses with <3 years of clinical experience (Mean = 3.67, *SD* = 0.42) had significantly higher job involvement than those with 3 to <5 years (Mean = 3.43, *SD* = 0.37; *p* < 0.05) and those with 5 to <10 years (Mean = 3.43, *SD* = 0.47; *p* < 0.05).

Furthermore, job involvement differed significantly according to ICU experience (*F* = 3.362, *p* = 0.020). The Scheffé post hoc test demonstrated that nurses with <3 years of ICU experience (Mean = 3.62, *SD* = 0.43) had significantly higher job involvement than those with 3 to <5 years of ICU experience (Mean = 3.41, *SD* = 0.42; *p* < 0.05). No significant differences in job involvement were observed based on gender, marital status, educational level, or religion (all *p* > 0.05).

### 3.2. Descriptive Statistics of the Study Variables

The descriptive statistics for the study variables are presented in Table 2. All variables and their subscales were evaluated using mean item scores (possible range: 1–5). The mean scores for job involvement, person-centred care, organisational communication satisfaction, and ethical climate were 3.53 (*SD* = 0.43), 3.63 (*SD* = 0.42), 3.41 (*SD* = 0.50), and 3.66 (*SD* = 0.49), respectively.

Among the subdomains of person-centred care, care processes had the highest mean item score (Mean = 3.81, *SD* = 0.45), whereas care environment had the lowest (Mean = 3.45, *SD* = 0.51). Among the subdomains of organisational communication satisfaction, vertical communication yielded the highest mean item score (Mean = 3.73, *SD* = 0.58), whereas organisational climate yielded the lowest (Mean = 2.88, *SD* = 0.70). Regarding the ethical climate subdomains, peers had the highest mean item score (Mean = 4.01, *SD* = 0.48), followed by managers (Mean = 3.87, *SD* = 0.75), while hospital had the lowest (Mean = 3.44, *SD* = 0.56).

### 3.3. Correlations Between Nurses’ Job Involvement, Person-Centred Care, Organisational Communication Satisfaction, and Ethical Climate

As shown in Table 3, job involvement was positively correlated with person-centred care (*r =* 0.637, *p <* 0.001), organisational communication satisfaction (*r =* 0.598, *p* < 0.001), and ethical climate (*r* = 0.536, *p <* 0.001). Among the subdomains of person-centred care, care environment (*r =* 0.587, *p <* 0.001) and care processes (*r =* 0.585, *p <* 0.001) showed the highest correlations with job involvement. Among the subdomains of organisational communication satisfaction, vertical communication (*r =* 0.538, *p <* 0.001) and horizontal communication (*r =* 0.525, *p <* 0.001) displayed the highest correlations with job involvement. Among the subdomains of ethical climate, physicians (*r =* 0.507, *p <* 0.001) and hospital (*r =* 0.479, *p <* 0.001) demonstrated the highest correlations with job involvement. The full correlation matrix for the 12 subdomain predictors and job involvement is provided in Table S2. Communication media was positively correlated with job involvement (*r* = 0.404), vertical communication (*r* = 0.676), horizontal communication (*r* = 0.571), and the hospital subdomain of ethical climate (*r* = 0.653).

### 3.4. Factors Associated with Nurses’ Job Involvement: Hierarchical Multiple Regression Analysis

Table 4 presents the results of the hierarchical multiple regression analysis examining factors associated with job involvement among ICU nurses (*n* = 217). The control variables and the subdomains of ethical climate, organisational communication satisfaction, and person-centred care were sequentially entered across four models to examine the additional variance explained by each set of variables. The Durbin–Watson statistic was 1.930. In the final model, VIF values ranged from 1.092 to 4.342, all below the conventional threshold of 10 [43,44].

Model 1 included gender, educational level, and total clinical experience as control variables. Gender and educational level were included even though neither was significantly associated with job involvement in the univariate analyses (Table 1); this was because they were associated with nurses’ job involvement and work engagement in previous studies [41,42]. Notably, age, total clinical experience, and ICU experience were each significantly associated with job involvement in the univariate analyses (Table 1). However, because these variables are closely interrelated, only total clinical experience, which broadly reflects overall professional experience, was entered into the model. Model 1 explained 7.3% of the variance in job involvement, *R*^2^ = 0.073; adjusted *R*^2^ = 0.047; *F*(6, 210) = 2.766, *p* = 0.013.

In Model 2, the five subdomains of ethical climate were added, significantly increasing the explained variance by 25.9 percentage points, Δ*R*^2^ = 0.259, Δ*F*(5, 205) = 15.939, *p* < 0.001. This model explained 33.3% of the total variance in job involvement (*R*^2^ = 0.333; adjusted *R*^2^ = 0.297).

In Model 3, the four subdomains of organisational communication satisfaction were added, producing an additional significant increase in explained variance of 11.4 percentage points, Δ*R*^2^ = 0.114, Δ*F*(4, 201) = 10.376, *p* < 0.001. This model explained 44.7% of the variance in job involvement (*R*^2^ = 0.447; adjusted *R*^2^ = 0.406).

In Model 4, the three subdomains of person-centred care were added, significantly increasing the explained variance by an additional 8.7 percentage points, Δ*R*^2^ = 0.087, Δ*F*(3, 198) = 12.308, *p* < 0.001. The final model explained 53.4% of the variance in job involvement, *R*^2^ = 0.534; adjusted *R*^2^ = 0.491; *F*(18, 198) = 12.596, *p* < 0.001. Vertical communication (β = 0.385, *p* < 0.001), horizontal communication (β = 0.209, *p* < 0.01), and the prerequisites subdomain of person-centred care (β = 0.191, *p* < 0.05) were independently and positively associated with job involvement. Conversely, the communication media subdomain (β = −0.166, *p* < 0.05) was significantly and negatively associated with job involvement. Among the statistically significant variables in the final model, vertical communication exhibited the largest absolute standardised regression coefficient.

## 4. Discussion

This study examined the associations of person-centred care, organisational communication satisfaction, and ethical climate with job involvement among ICU nurses in South Korea. In the final model, vertical communication, horizontal communication, and the prerequisites subdomain of person-centred care were independently and positively associated with job involvement, whereas communication media satisfaction showed a negative adjusted association. The final model explained 53.4% of the variance in job involvement (*R*^2^ = 0.534; adjusted *R*^2^ = 0.491). Adding the person-centred care subdomains increased *R*^2^ by 8.7 percentage points (Δ*R*^2^ = 0.087) compared with the model containing only the control variables, ethical climate, and organisational communication satisfaction. These findings draw attention to communication within nursing organisations and the professional attributes forming the basis of person-centred practice, while also illustrating the importance of distinguishing bivariate relationships from associations adjusted for other variables.

Satisfaction with vertical communication—a subdomain of organisational communication satisfaction encompassing communication with superiors (including nurse managers, charge nurses, and senior nurses [39])—had the largest positive standardised coefficient in the final model. ICU nursing requires specialised knowledge, continuous assessment, and clinical judgement tailored to individual patients. Much of this expertise is developed at the bedside through guidance and feedback from experienced nurses [45,46]. Previous research has highlighted the relevance of hierarchy and leadership to nurses’ opportunities to express their views [47,48]. In South Korea, Lee et al. [49] identified hierarchical relationships, power differences, and seniority as barriers to speaking up, while supportive managers and constructive responses encouraged nurses to express concerns. Hierarchical unit culture, power distance orientation, and perceived futility about speaking up have also been associated with nurses’ silence behaviour [50]. Thus, in the context of ICUs, the way in which superiors respond when nurses raise concerns or reveal uncertainty may be central to whether such disclosure becomes an opportunity for learning or a source of pressure. Nurses who are satisfied with communication with their superiors may perceive greater opportunities to have their clinical judgement heard and considered [18,47]. Such recognition could help nurses experience their job as one in which they can develop and exercise their expertise, offering a potential explanation for the association between satisfaction with vertical communication and job involvement. However, these mechanisms require further investigation, given that this study did not directly examine superiors’ responses or support for learning.

Satisfaction with horizontal communication—another subdomain of organisational communication satisfaction concerning everyday exchanges with peers at a similar level (with whom ICU nurses share responsibility for patient care across shifts [39])—was also independently and positively associated with job involvement. Because ICU nurses often work side by side in shared spaces, their practice is readily visible to peers. This visibility can create opportunities for timely assistance and informal feedback, although feedback may also be experienced as criticism that discourages nurses from asking questions [51,52]. Research on nurse–nurse collaboration and peer learning has drawn attention to these interpersonal dimensions of ICU nursing practice [53,54]. A qualitative systematic review found that newly qualified critical care nurses valued sharing experiences with peers who understood their difficulties [55]. Although the review focused on nurses transitioning into critical care rather than on job involvement, it illustrates how peer interactions can provide vital support in a demanding clinical setting. Satisfactory communication with peers may make it easier for nurses to seek advice, discuss their clinical reasoning, and receive feedback as respected members of the team. This could strengthen their sense of contributing to patient care and deepen their psychological identification with their job. However, this study did not directly examine feedback experiences or nurses’ sense of contribution as mechanisms potentially underlying this association.

The prerequisites subdomain of person-centred care was also positively associated with job involvement. This subdomain encompasses professional competence, interpersonal skills, commitment to care, self-awareness, and clarity of beliefs and values [38,56]. van der Cingel and Brouwer [57] emphasised the integration of nursing knowledge and moral values into everyday practice in their discussion of nursing professional identity. Such integration is particularly relevant to ICU nursing, where specialised knowledge and technical skills must be applied to the needs of individual patients, many of whom cannot readily communicate their preferences or concerns [15,16]. Nurses’ professional competence has been linked not only to their confidence in their clinical abilities but also to the meaning they attach to using those abilities and to their work engagement [58,59]. Recognising subtle changes in a patient’s condition, anticipating needs that the patient cannot express, and providing care that respects the patient’s dignity may be important ways in which ICU nurses enact their understanding of good nursing [16,30]. Nurses who perceive themselves as able to practise in accordance with their professional knowledge and values may therefore experience their job as an expression of what matters to them. Such experiences may be one way in which the prerequisites of person-centred care relate to job involvement.

This interpretation concerns the professional attributes underpinning person-centred practice (prerequisites). By contrast, care environment and care processes, which showed the strongest bivariate correlations with job involvement among the person-centred care subdomains (Table 3), did not show statistically significant adjusted associations in the final model. This pattern may reflect shared variance among the person-centred care subdomains and with the other predictors. Consequently, it should not be taken to indicate that the prerequisites of person-centred care are more important than the other subdomains [60,61]. Notably, however, ‘commitment to care’ within the prerequisites subdomain may overlap conceptually with job involvement, which could partly account for the observed association. For example, the commitment-to-care items in the questionnaire concern efforts to provide high-quality and evidence-informed care, become acquainted with patients and families to provide holistic care, and improve care experiences [37]. Similarly, job involvement items concerning efforts to improve work performance, personal responsibility, and willingness to contribute beyond usual duties share an emphasis on effort and dedication [36]. However, the underlying focus of the two constructs differs: the former concern care delivery [37], whereas the latter also address the personal importance of the job, growth, achievement, and contribution to the hospital [36]. The model used the full 13-item prerequisites score rather than a separate commitment-to-care score. Thus, although the association between prerequisites and job involvement may partly reflect overlapping item content, the extent of this overlap cannot be determined from the present analyses.

Satisfaction with communication media showed a negative adjusted association with job involvement despite a positive bivariate correlation. This subdomain includes satisfaction with meetings and the perceived adequacy of information-sharing systems, participation in informal gatherings and their perceived usefulness, and exposure to workplace rumours [39]. It therefore encompasses a range of communication experiences, rather than measuring satisfaction with communication channels alone. The full matrix (Table S2) shows that communication media was correlated with vertical communication (*r* = 0.676), horizontal communication (*r* = 0.571), and the hospital subdomain of ethical climate (*r* = 0.653), alongside its positive correlation with job involvement (*r* = 0.404). The negative final-model coefficient (β = −0.166) describes its association with job involvement after adjustment for the other included predictors. Shared variance and a possible suppression effect may contribute to this sign reversal [60,61], but the correlation matrix alone does not demonstrate suppression. The coefficient should not be interpreted as evidence that communication media satisfaction reduces job involvement or that reducing communication would be beneficial. The relatively low internal consistency of this subdomain (α = 0.62) further warrants caution when interpreting this finding.

Although the overall ethical climate score was positively correlated with job involvement, none of its subdomains retained a statistically significant adjusted association in the final model. In Model 2, only the physicians subdomain was significantly associated with job involvement. However, its coefficient decreased after organisational communication satisfaction was added in Model 3 and was no longer statistically significant after the person-centred care subdomains were entered in Model 4. This pattern may reflect shared variance with the additional predictors [60,61], although the present analyses do not establish what caused this attenuation. Therefore, the findings from the model should not be interpreted as evidence that ethical climate is unrelated to job involvement. The conceptual and empirical overlap between ethical climate and communication is also relevant: vertical communication was correlated with the hospital (*r* = 0.767) and managers (*r* = 0.749) subdomains of ethical climate (Table S2). These relationships reinforce the need to interpret individual coefficients in the context of the other predictors.

### 4.1. Implications

This study contributes to the understanding of ICU nurses’ job involvement by examining person-centred care, organisational communication satisfaction, and ethical climate within a single analytical framework. The findings suggest that nurses’ psychological identification with their job is concurrently associated with communication within the nursing organisation and the professional attributes they bring to person-centred practice. The additional variance accounted for by the person-centred care subdomains supports the idea that person-centred practice should be considered alongside organisational communication and ethical climate. For nurse managers, the findings suggest that communication with superiors and peers warrants attention alongside the professional attributes forming the prerequisites of person-centred practice. Potential applications for vertical communication include structured opportunities for staff nurses to raise clinical concerns with superiors and receive constructive feedback, as well as two-way, open communication channels through which nurse managers share their vision with staff nurses. For horizontal communication, peer discussions of challenging care situations and collaborative problem-solving warrant evaluation. Additionally, professional development could incorporate reflection on interpersonal skills, self-awareness, and professional beliefs and values, alongside technical competence. Because these approaches were not evaluated in this study, they should be regarded as possibilities for development and testing rather than established strategies for increasing job involvement. Prospective, longitudinal, and interventional studies are required to determine whether changes in these organisational and professional conditions lead to changes in job involvement.

### 4.2. Limitations

Several limitations should be considered. First, the cross-sectional design precludes conclusions about causality or temporal ordering. Nurses who identify more strongly with their job may perceive their communication experiences, ethical climate, and professional capabilities more favourably; reciprocal relationships are also possible.

Second, convenience sampling from only three hospitals limits generalisability. Differences in hospital characteristics, organisational culture, ICU specialties, patient acuity, and working environments may have contributed to the observed associations. The findings may not apply to ICUs operating under different organisational, clinical, or cultural conditions, including healthcare systems in which communication between nurse managers and staff nurses is less hierarchical. Hospital-specific participant counts were not reported because of confidentiality concerns, limiting assessment of the balance of recruitment across hospitals. Hospital membership and potential within-hospital dependence were not accounted for in the models. Consequently, the possibility of correlated observations within hospitals cannot be excluded. If such dependence was present, standard errors may have been underestimated, confidence intervals may have been too narrow, and statistical significance may have been overstated. The small number of hospitals also constrains conventional cluster-robust inference and estimation of between-hospital variability.

Third, all variables were measured using self-report questionnaires at one time point. Shared response tendencies and social desirability may have introduced common method bias [62]; its magnitude and direction were not established. Self-reported prerequisites should not be equated with independently assessed professional competence or care quality. Fourth, the measures include related organisational and interpersonal content, and commitment to care may overlap with the effort and dedication captured by job involvement. Such overlap and shared variance complicate interpretation of the adjusted coefficients. Internal consistency was relatively low for communication media (α = 0.62) and at the lower end of the generally accepted range for the patients subdomain of ethical climate (α = 0.70), warranting caution about measurement precision and the corresponding associations.

Fifth, multiple group comparisons, correlations, and regression coefficients were examined without a study-wide multiplicity adjustment. Scheffé tests addressed post hoc ANOVA comparisons only. Some statistically significant findings may therefore reflect chance and require replication. Sixth, staffing adequacy, workload, and leadership practices were not separately included as organisational covariates. These factors could be associated with both the predictors and job involvement, leaving residual confounding possible. Furthermore, excluding age and ICU experience because of their overlap with total clinical experience, and omitting other covariates based on non-significant univariate comparisons, does not eliminate the possibility of residual confounding.

Finally, incremental explained variance depends on the order of block entry and should not be interpreted as an order-independent measure of importance. Longitudinal studies using multiple data sources and a broader sample of hospitals are needed to clarify the direction and generalisability of the associations. Interventional studies are needed to evaluate the proposed organisational applications.

## 5. Conclusions

Satisfaction with vertical and horizontal communication and the prerequisites of person-centred practice were independently and positively associated with ICU nurses’ job involvement. Conversely, communication media satisfaction exhibited a negative adjusted association that requires cautious interpretation. Overall ethical climate was positively correlated with job involvement, but none of its subdomains showed a statistically significant adjusted association in the final model. These findings highlight communication with superiors and peers, alongside the professional capabilities and values forming the basis of person-centred practice, as relevant to understanding nurses’ job involvement. Suggestions concerning communication and professional development represent potential organisational applications that require further evaluation. Longitudinal studies are needed to clarify the direction of the associations, and interventional research is needed to evaluate potential organisational approaches.

## Figures and Tables

**Table 1:** Differences in job involvement according to nurses’ general characteristics (*n* = 217).

Characteristics	Categories	*n* (%)		Job Involvement				
				Mean	(*SD*)	*t* or *F*	*p*	*Scheffé Post Hoc Test*
Gender	Female	177	(81.6)	3.52	(0.44)	−0.755	0.451	
	Male	40	(18.4)	3.58	(0.40)			
Age (years)	≤25 ^a^	66	(30.4)	3.69	(0.38)	4.586	0.004	b < a
	26–30 ^b^	100	(46.1)	3.46	(0.44)			
	31–35 ^c^	32	(14.7)	3.43	(0.38)			
	≥36 ^d^	19	(8.8)	3.55	(0.46)			
Marital status	Married	34	(15.7)	3.50	(0.49)	−0.514	0.608	
	Unmarried	183	(84.3)	3.54	(0.42)			
Educational level	Associate degree	29	(13.4)	3.61	(0.34)	0.650	0.521	
	Bachelor’s degree	175	(80.6)	3.52	(0.44)			
	Graduate degree or higher	13	(6.0)	3.57	(0.43)			
Religion	Yes	54	(24.9)	3.51	(0.45)	−0.337	0.736	
	No	163	(75.1)	3.54	(0.42)			
Total clinical experience (years)	<3 ^a^	78	(35.9)	3.67	(0.42)	5.112	0.002	b, c < a
	3 to <5 ^b^	64	(29.5)	3.43	(0.37)			
	5 to <10 ^c^	44	(20.3)	3.43	(0.47)			
	≥10 ^d^	31	(14.3)	3.52	(0.42)			
ICU experience (years)	<3 ^a^	102	(47.0)	3.62	(0.43)	3.362	0.020	b < a
	3 to <5 ^b^	57	(26.3)	3.41	(0.42)			
	5 to <10 ^c^	42	(19.4)	3.53	(0.41)			
	≥10 ^d^	16	(7.4)	3.43	(0.42)			

Note. *SD* = standard deviation; *t* = independent-sample *t*-test; *F* = one-way analysis of variance (ANOVA). a, b, c, d: Groups from *Scheffé post hoc test* (*p* < 0.05).

**Table 2:** Levels of job involvement, person-centred care, organisational communication satisfaction, and ethical climate (*n* = 217).

Variables	Mean (*SD*)	Min–Max
**Job involvement**	3.53 (0.43)	1.89–4.78
**Person-centred care**	3.63 (0.42)	2.47–4.96
Care processes	3.81 (0.45)	2.44–5.00
Prerequisites	3.72 (0.42)	2.69–5.00
Care environment	3.45 (0.51)	1.86–4.95
**Organi** **s** **ational communication satisfaction**	3.41 (0.50)	1.21–4.67
Vertical communication	3.73 (0.58)	1.00–5.00
Communication media	3.46 (0.55)	1.67–5.00
Horizontal communication	3.37 (0.62)	1.00–5.00
Organisational climate	2.88 (0.70)	1.00–5.00
**Ethical climate**	3.66 (0.49)	1.69–4.92
Peers	4.01 (0.48)	2.50–5.00
Managers	3.87 (0.75)	1.00–5.00
Patients	3.65 (0.47)	2.25–5.00
Physicians	3.45 (0.74)	1.00–5.00
Hospital	3.44 (0.56)	1.00–4.83

Note. *SD* = standard deviation; Min–Max = observed minimum and maximum scores. All scores were calculated as the mean of item scores (possible range: 1–5). Within each variable, subdomains are listed in descending order of mean score.

**Table 3:** Correlations of job involvement with person-centred care, organisational communication satisfaction, ethical climate, and their subdomains (*n* = 217).

	Job Involvement	
	*r*	*p*
**Person-centred care**	0.637	<0.001
Care environment	0.587	<0.001
Care processes	0.585	<0.001
Prerequisites	0.530	<0.001
**Organi** **s** **ational communication satisfaction**	0.598	<0.001
Vertical communication	0.538	<0.001
Horizontal communication	0.525	<0.001
Organisational climate	0.481	<0.001
Communication media	0.404	<0.001
**Ethical climate**	0.536	<0.001
Physicians	0.507	<0.001
Hospital	0.479	<0.001
Peers	0.398	<0.001
Patients	0.353	<0.001
Managers	0.352	<0.001

Note. *r* = Pearson’s correlation coefficient. Within each variable, subdomains are listed in descending order of *r*.

**Table 4:** Hierarchical multiple regression analysis of factors associated with nurses’ job involvement (*n* = 217).

	Model 1		Model 2			Model 3			Model 4			
	*B*	β	*B*	β		*B*	β		*B*	β		95% CI for *B*
**Constant**	3.576		2.019			1.892			1.106			[0.597, 1.616]
**Gender**												
Male	0.057	0.052	0.001	0.001		0.035	0.031		0.058	0.053		[−0.052, 0.168]
**Educational level**												
Associate degree	−0.035	−0.028	−0.170	−0.135		−0.256	−0.203		−0.183	−0.145		[−0.423, 0.057]
Bachelor’s degree	−0.090	−0.083	−0.153	−0.141		−0.196	−0.180		−0.161	−0.149		[−0.368, 0.045]
**Total clinical experience (years)**												
<3	0.165	0.185	0.080	0.090		0.060	0.067		0.130	0.146		[−0.030, 0.290]
3 to <5	−0.072	−0.077	−0.029	−0.030		−0.047	−0.050		0.047	0.051		[−0.113, 0.208]
5 to <10	−0.077	−0.073	−0.056	−0.053		−0.046	−0.043		−0.006	−0.005		[−0.172, 0.161]
**Ethical climate**												
Physicians			0.196	0.340	***	0.120	0.207	*	0.076	0.131		[−0.016, 0.167]
Peers			0.102	0.113		0.034	0.037		0.024	0.027		[−0.107, 0.155]
Hospital			0.070	0.092		−0.085	−0.112		−0.123	−0.161		[−0.272, 0.026]
Patients			0.073	0.079		0.060	0.065		−0.024	−0.026		[−0.152, 0.104]
Managers			0.014	0.025		−0.059	−0.104		−0.054	−0.095		[−0.140, 0.032]
**Organisational communication satisfaction**												
Vertical communication						0.279	0.374	***	0.287	0.385	***	[0.139, 0.436]
Horizontal communication						0.217	0.316	***	0.144	0.209	**	[0.046, 0.242]
Organisational climate						0.072	0.118		0.034	0.055		[−0.058, 0.125]
Communication media						−0.114	−0.146		−0.130	−0.166	*	[−0.247, −0.013]
**Person-centred care**												
Prerequisites									0.196	0.191	*	[0.042, 0.351]
Care processes									0.148	0.156		[−0.013, 0.309]
Care environment									0.108	0.128		[−0.043, 0.260]
*R*^2^ (Δ*R*^2^)	0.073 (0.073)		0.333 (0.259)			0.447 (0.114)			0.534 (0.087)			
Adjusted *R*^2^	0.047		0.297			0.406			0.491			
*F* (*p*)	2.766 (0.013)		9.291 (<0.001)			10.827 (<0.001)			12.596 (<0.001)			
Δ*F* (*p*)	2.766 (0.013)		15.939 (<0.001)			10.376 (<0.001)			12.308 (<0.001)			
Durbin–Watson									1.930			

Note. *B* = unstandardised regression coefficient; β = standardised regression coefficient; *R*^2^ = coefficient of determination; Δ*R*^2^ = change in *R*^2^; ΔF = change in *F* statistic; CI = confidence interval for *B*. Degrees of freedom for *F* were (6, 210), (11, 205), (15, 201), and (18, 198) for Models 1–4, respectively; those for ΔF were (6, 210), (5, 205), (4, 201), and (3, 198), respectively. Reference groups: gender = female; educational level = graduate degree or higher; total clinical experience = ≥10 years. Within each block, subdomains are listed in descending order of β in the model in which they were entered. * *p* < 0.05, ** *p* < 0.01, *** *p* < 0.001.

## Data Availability

The data presented in this study are available on reasonable request from the corresponding author. The data are not publicly available due to privacy and ethical restrictions.

## Supplementary Materials

The following supporting information can be downloaded at: https://www.mdpi.com/article/10.3390/healthcare14213824/s1, Table S1: STROBE checklist for cross-sectional studies; Table S2: Pearson correlation matrix for job involvement and the subdomains entered into the regression models (*n* = 217).

## Abbreviations

The following abbreviations are used in this manuscript:

ICU	Intensive care unit
STROBE	Strengthening the Reporting of Observational Studies in Epidemiology
K-PCPI-S	Korean version of the Person-Centred Practice Inventory–Staff
K-HECS	Korean version of the Hospital Ethical Climate Survey
ANOVA	Analysis of variance
VIF	Variance inflation factor
CI	Confidence interval
*SD*	Standard deviation
