# Supplementary material for: Associations of Person-Centred Care, Organisational Communication Satisfaction, and Ethical Climate with Job Involvement Among Intensive Care Unit Nurses in South Korea: A Cross-Sectional Study
Source: Healthcare (Basel). 2026 Nov 9;14(21):3824. doi: 10.3390/healthcare14213824 (PMC13762664; doi:10.3390/healthcare14213824)
Supplement: Supplementary file 1 [file healthcare-14-03824-s001.zip › 2026.11.05 S1 Supplementary_Table STROBE_Cross_Sectional_Checklist.pdf]

Table S1. STROBE checklist for cross-sectional studies

Manuscript title: Associations of Person-Centred Care, Organisational Communication Satisfaction, and Ethical Climate with Job Involvement Among Intensive Care Unit Nurses in South Korea: A Cross-Sectional Study

Study information: 217 intensive care unit nurses in South Korea; cross-sectional study; hierarchical multiple regression analysis.

Reporting status: Page numbers refer to the submitted manuscript. Each item is marked Reported, Partially reported, Not reported, Not performed or Not applicable.

Title and abstract and Introduction

Section / topic	Item	Official STROBE recommendation	Manuscript page	Reporting status and description
Title and abstract	1(a)	Indicate the study's design with a commonly used term in the title or the abstract	1	Reported. Title: "A Cross-Sectional Study"; abstract Methods: "cross-sectional descriptive correlational study".
Title and abstract	1(b)	Provide in the abstract an informative and balanced summary of what was done and what was found	1–2	Reported. Structured abstract summarises objectives, design, sample (n = 217), analysis, key estimates (r, R^2, ΔR^2, β) and cautions (negative adjusted coefficient for communication media; need for longitudinal research). Setting (three general hospitals) and data collection period (August 2025) stated in abstract Methods.
Background/ rationale	2	Explain the scientific background and rationale for the investigation being reported	2–3	Reported. Introduction covers ICU context, job involvement, person-centred care, organisational communication satisfaction and ethical climate, and the evidence gap, stated as few studies having examined these factors simultaneously in relation to job involvement among ICU nurses (p. 3).
Objectives	3	State specific objectives, including any prespecified hypotheses	3	Reported. The study objective is stated in the final paragraph of the Introduction (p. 3). No formal hypotheses were prespecified.

Methods

Section / topic	Item	Official STROBE recommendation	Manuscript page	Reporting status and description
Study design	4	Present key elements of study design early in the paper	1, 4	Reported. Abstract and Section 2.1 (first sentence): cross-sectional descriptive correlational design, reported in accordance with STROBE.
Setting	5	Describe the setting, locations, and relevant dates, including periods of recruitment, exposure, follow-up, and data collection	4	Reported. Three general hospitals (>200 beds) in Seoul, Gangwon and Gyeonggi; data collected 1–31 August 2025 (Section 2.1, p. 4). Follow-up not applicable (single time point).

Section / topic	Item	Official STROBE recommendation	Manuscript page	Reporting status and description
Participants	6(a)	Give the eligibility criteria, and the sources and methods of selection of participants	4	Reported. Inclusion: ICU staff nurses providing written consent; exclusion: <6 months' ICU experience (still in orientation and training) and unit managers (roles and work environments differ from those of staff nurses), with rationale referenced to previous research; convenience sampling. Before obtaining written informed consent, the first author explained the study's purpose and informed potential participants that participation was voluntary and that they could withdraw at any time without repercussions (Section 2.1, p. 4). The first author visited the participating ICUs in person to distribute the questionnaires; completed questionnaires were sealed in envelopes and collected by the first author (Section 2.1, p. 4).
Variables	7	Clearly define all outcomes, exposures, predictors, potential confounders, and effect modifiers. Give diagnostic criteria, if applicable	4–6, 9	Reported. Outcome (job involvement), predictors (person-centred care, organisational communication satisfaction and ethical climate subdomains) and control variables (gender, education, total clinical experience) defined in Sections 2.2–2.3; the control variables entered in the regression are specified in Section 3.4 (p. 9). No effect modifiers examined; diagnostic criteria not applicable.
Data sources/ measurement	8*	For each variable of interest, give sources of data and details of methods of assessment (measurement). Describe comparability of assessment methods if there is more than one group	4–5	Reported. Self-administered questionnaires (p. 4); instruments, item numbers, response scales, scoring, reverse coding and Cronbach's α (Sections 2.2.1–2.2.5, pp. 4–5). Single group, so comparability across groups not applicable.
Bias	9	Describe any efforts to address potential sources of bias	4, 13–14	Partially reported. Sealed envelopes for confidentiality (p. 4); convenience sampling limiting generalisability, possible within-hospital dependence, social desirability, common method bias and residual confounding acknowledged in Limitations (pp. 13–14).
Study size	10	Explain how the study size was arrived at	4	Reported. G*Power 3.1.9 for multiple regression (α = 0.05, power = 0.90, f^2 = 0.15): minimum 202; 240 distributed allowing ~15% non-response or incomplete responses (Section 2.1, p. 4). The calculation assumed 23 candidate predictors (the 18 final-model predictors plus age, marital status and religion dummy variables); a repeat calculation with 18 predictors yielded a minimum of 183, which the final sample of 217 exceeded. Both calculations apply to the overall model, not to individual coefficients (Section 2.1, p. 4).
Quantitative variables	11	Explain how quantitative variables were handled in the analyses. If applicable, describe which groupings were chosen and why	6–7	Reported. Scales analysed as mean item scores (1–5); categorical variables dummy-coded with stated reference groups (Section 2.3, p. 6); age and experience groupings shown in Table 1 (p. 7). Age and experience were grouped for descriptive and group-comparison analyses, following categorical reporting approaches in previous nursing ethics studies and the categories used in previous studies of Korean ICU nurses, with a minor modification to the upper age boundary (Section 2.2.1, p. 4).

Statistical methods

Section / topic	Item	Official STROBE recommendation	Manuscript page	Reporting status and description
Statistical methods	12(a)	Describe all statistical methods, including those used to control for confounding	5–6, 9	Reported. Section 2.3 (pp. 5–6): descriptive statistics, Cronbach's α reliability analysis, independent-sample t-tests, ANOVA with Scheffé post hoc tests, Pearson's r, hierarchical multiple regression, dummy coding with stated reference categories, VIF and Durbin–Watson criteria, B with 95% CIs, two-sided α = 0.05, SPSS 29.0.2.0. The four-block entry sequence, the control variables and the rationale for their selection (gender and educational level based on previous studies; total clinical experience only, to minimise conceptual overlap with age and ICU experience; marital status and religion not retained because of non-significant univariate differences) are described in Section 2.3 (p. 6) and Section 3.4 (p. 9). No study-wide adjustment for multiple testing was applied; the Scheffé adjustment was limited to post hoc ANOVA comparisons (Section 2.3, p. 6).
Statistical methods	12(b)	Describe any methods used to examine subgroups and interactions	—	Not performed. No subgroup or interaction analyses were conducted; group comparisons in Table 1 are univariate descriptive analyses.
Statistical methods	12(c)	Explain how missing data were addressed	4, 6	Reported. Twelve questionnaires were excluded before analysis because of item non-response or selection of the same response option for every item (Section 2.1, p. 4). The 217 questionnaires included in the analysis contained no missing item responses; therefore, no imputation was required. Scale and subscale scores were calculated as the mean of the item responses within the respective scale or subscale, and all 217 participants were included in the regression analysis (Section 2.3, p. 6).
Statistical methods	12(d)	If applicable, describe analytical methods taking account of sampling strategy	4, 6, 13–14	Not performed. Convenience sampling from three hospitals (Section 2.1, p. 4); data were analysed as a pooled sample, without hospital membership as a covariate or multilevel/cluster-adjusted analysis (Section 2.3, p. 6). Limitations (pp. 13–14) acknowledge possible within-hospital dependence, with potentially underestimated standard errors and overstated statistical significance.
Statistical methods	12(e)	Describe any sensitivity analyses	—	Not performed. No sensitivity analyses were conducted.

Results

Section / topic	Item	Official STROBE recommendation	Manuscript page	Reporting status and description
Participants	13(a)*	Report numbers of individuals at each stage of study—eg numbers potentially eligible, examined for eligibility, confirmed eligible, included in the study, completing follow-up, and analysed	4	Partially reported. 240 distributed, 229 returned (95.4%) and 217 analysed (p. 4). Number of potentially eligible nurses in the participating ICUs not reported.

Section / topic	Item	Official STROBE recommendation	Manuscript page	Reporting status and description
Participants	13(b)*	Give reasons for non-participation at each stage	4	Partially reported. 12 excluded (item non-response or the same response option for every item); no reasons given for the 11 non-returned questionnaires.
Participants	13(c)*	Consider use of a flow diagram	—	Not performed. No flow diagram; the single-stage recruitment is described in text (p. 4).
Descriptive data	14(a)*	Give characteristics of study participants (eg demographic, clinical, social) and information on exposures and potential confounders	6–8	Reported. Table 1 (p. 7; "Differences in job involvement according to nurses' general characteristics"): gender, age, marital status, education, religion, total and ICU experience; Table 2 (p. 8): descriptive statistics for all exposure variables and subdomains.
Descriptive data	14(b)*	Indicate number of participants with missing data for each variable of interest	6	Reported. Section 2.3 (p. 6) states that the 217 analysed questionnaires contained no missing item responses, so no participant had missing data for any variable of interest. All tables use n = 217.
Outcome data	15*	Report numbers of outcome events or summary measures	6–8	Reported. Job involvement summary measures: mean 3.53 (SD 0.43), range 1.89–4.78, n = 217 (Table 2, p. 8); by participant characteristics in Table 1 (p. 7).

Main results and other analyses

Section / topic	Item	Official STROBE recommendation	Manuscript page	Reporting status and description
Main results	16(a)	Give unadjusted estimates and, if applicable, confounder-adjusted estimates and their precision (eg, 95% confidence interval). Make clear which confounders were adjusted for and why they were included	8–10	Reported. Unadjusted: correlations (Table 3, p. 9); adjusted: B, β, R^2 and ΔR^2 (Table 4, p. 10) with 95% CIs for Model 4 coefficients (Table 4, p. 10; stated in Section 2.3); confounder rationale in Section 3.4 (p. 9).
Main results	16(b)	Report category boundaries when continuous variables were categorized	7, 10	Reported. Category boundaries for age and clinical/ICU experience in Table 1; categories and reference groups in Table 4 and note. Continuous scale scores were not categorised.
Main results	16(c)	If relevant, consider translating estimates of relative risk into absolute risk for a meaningful time period	—	Not applicable. Continuous outcome analysed with linear regression; no relative risk estimated.
Other analyses	17	Report other analyses done—eg analyses of subgroups and interactions, and sensitivity analyses	6–7, 9–10	Reported. Group comparisons by characteristics (Table 1, with Scheffé tests) and regression diagnostics (VIF range and Durbin–Watson statistic in Section 3.4, p. 9; Durbin–Watson also in Table 4, p. 10). No subgroup, interaction or sensitivity analyses, consistent with Methods.

Discussion and other information

Section / topic	Item	Official STROBE recommendation	Manuscript page	Reporting status and description
Key results	18	Summarise key results with reference to study objectives	10	Reported. First paragraph of Discussion summarises key results with reference to the study aim.

Section / topic	Item	Official STROBE recommendation	Manuscript page	Reporting status and description
Limitations	19	Discuss limitations of the study, taking into account sources of potential bias or imprecision. Discuss both direction and magnitude of any potential bias	13–14	Partially reported. Section 4.2 covers causality (possible reciprocal relationships), convenience sampling limiting generalisability, unaccounted within-hospital dependence (standard errors possibly underestimated and significance overstated), social desirability and common method bias (magnitude and direction acknowledged as not established), self-reported rather than independently assessed competence, conceptual overlap and shared variance among measures, low internal consistency for communication media (α = 0.62), the absence of study-wide multiplicity adjustment, residual confounding (staffing adequacy, workload and leadership practices not included; exclusion of age and ICU experience) and the order-dependence of incremental variance. The likely direction of bias is stated only for within-hospital dependence; the magnitude of potential biases is not quantified.
Interpretation	20	Give a cautious overall interpretation of results considering objectives, limitations, multiplicity of analyses, results from similar studies, and other relevant evidence	10–14	Reported. Cautious interpretation (e.g., “offering a potential explanation”; mechanisms not directly examined), caution on the non-significant adjusted associations for care environment and care processes despite their strong bivariate correlations (p. 12), the negative adjusted coefficient for communication media and the attenuated ethical climate subdomain associations (pp. 12–13), and comparison with previous studies. Multiplicity is acknowledged in Limitations: no study-wide adjustment was applied, so some significant findings may reflect chance and require replication (p. 14).
Generalisability	21	Discuss the generalisability (external validity) of the study results	13	Reported. Limitations (Section 4.2) note that convenience sampling from three hospitals limits generalisability to other ICU settings and to healthcare systems with different organisational cultures.
Funding	22	Give the source of funding and the role of the funders for the present study and, if applicable, for the original study on which the present article is based	15	Reported. Funding: Hallym University Research Fund, 2026 (HRF-202602-007) (p. 15); Conflicts of Interest states that the funders had no role in study design, data collection, analysis, interpretation, writing or the decision to publish (p. 15).

*Give information separately for exposed and unexposed groups.

Note: An Explanation and Elaboration article discusses each checklist item and gives methodological background and published examples of transparent reporting. The STROBE checklist is best used in conjunction with this article (freely available on the Web sites of PLoS Medicine at http://www.plosmedicine.org/, Annals of Internal Medicine at http://www.annals.org/, and Epidemiology at http://www.epidem.com/). Information on the STROBE Initiative is available at www.strobe-statement.org.

Source: STROBE Statement—Checklist of items that should be included in reports of cross-sectional studies. https://www.strobe-statement.org/download/strobe-checklist-cross-sectional-studies-pdf (accessed 17 October 2026).
