# Supplementary material for: Associations of Person-Centred Care, Organisational Communication Satisfaction, and Ethical Climate with Job Involvement Among Intensive Care Unit Nurses in South Korea: A Cross-Sectional Study
Source: Healthcare (Basel). 2026 Nov 9;14(21):3824. doi: 10.3390/healthcare14213824 (PMC13762664; doi:10.3390/healthcare14213824)
Supplement: Supplementary file 1 [file healthcare-14-03824-s001.zip › 2026.11.05 S2 Supplementary_Table_S2.pdf]

Table S2. Pearson correlation matrix for job involvement and the subdomains entered into the regression models (n = 217)

Variable	1	2	3	4	5	6	7	8	9	10	11	12	13
1	—												
2	0.530***	—											
3	0.587***	0.688***	—										
4	0.585***	0.694***	0.720***	—									
5	0.538***	0.338***	0.562***	0.539***	—								
6	0.525***	0.456***	0.546***	0.492***	0.482***	—							
7	0.481***	0.380***	0.573***	0.462***	0.523***	0.615***	—						
8	0.404***	0.383***	0.559***	0.472***	0.676***	0.571***	0.511***	—					
9	0.398***	0.309***	0.421***	0.492***	0.635***	0.404***	0.313***	0.554***	—				
10	0.353***	0.336***	0.416***	0.545***	0.562***	0.302***	0.303***	0.459***	0.650***	—			
11	0.352***	0.276***	0.445***	0.428***	0.749***	0.347***	0.402***	0.509***	0.484***	0.483***	—		
12	0.479***	0.414***	0.626***	0.561***	0.767***	0.534***	0.642***	0.653***	0.537***	0.485***	0.649***	—	
13	0.507***	0.352***	0.575***	0.511***	0.616***	0.531***	0.553***	0.537***	0.442***	0.342***	0.439***	0.721***	—

1 = Job involvement; 2 = PCC prerequisites; 3 = PCC care environment; 4 = PCC care processes; 5 = Vertical communication; 6 = Horizontal communication; 7 = Organisational climate; 8 = Communication media; 9 = Ethical climate—peers; 10 = Ethical climate—patients; 11 = Ethical climate—managers; 12 = Ethical climate—hospital; 13 = Ethical climate—physicians.

Note. PCC = person-centred care. Values are Pearson's r; the lower triangle is shown because the matrix is symmetric. All correlations use 217 complete observations. Tests are two-sided and unadjusted for multiple comparisons. *$p < 0.05$, **$p < 0.01$, ***$p < 0.001$.
